# Improved Ozonation Efficiency for Polymerization Mother Liquid from Polyvinyl Chloride Production Using Tandem Reactors

**DOI:** 10.3390/molecules24244436

**Published:** 2019-12-04

**Authors:** Zhiyong Yang, Penglei Wang, Yagang Zhang, Xingjie Zan, Wenjuan Zhu, Yingfang Jiang, Letao Zhang, Akram Yasin

**Affiliations:** 1 Xinjiang Technical Institute of Physics and Chemistry, Chinese Academy of Sciences, Urumqi 830011, China; yangzy@ms.xjb.ac.cn (Z.Y.); wangpl@mail.dlut.edu.cn (P.W.); zhuwj@ms.xjb.ac.cn (W.Z.); jiangyf@ms.xjb.ac.cn (Y.J.); zhanglt@ms.xjb.ac.cn (L.Z.); akram@ms.xjb.ac.cn (A.Y.); 2 University of Chinese Academy of Sciences, Beijing 100049, China; 3 Department of Chemical and Environmental Engineering, Xinjiang Institute of Engineering, Urumqi 830023, China; 4 School of Materials and Energy, University of Electronic Science and Technology of China, Chengdu 611731, China

**Keywords:** polymerization mother liquid, ozonation utilization efficiency, tandem reactor, COD

## Abstract

Polymerization mother liquid (PML) is one of the main sources of wastewater in the chlor-alkali industry. The effective degradation of the PML produced in PVC polymerization using three or five ozone reactors in tandem was designed with a focus on improving the ozonation efficiency. The ozonation efficiency of the tandem reactors for the degradation of PML, along with the effect of ozone concentration, the number of reactors utilized in series, and the reaction time on the chemical oxygen demand (COD) removal were investigated in detail. The results showed that the COD removal increased as the ozone concentration was increased from 10.6 to 60 mg·L^−1^, achieving 66.4% COD removal at ozone concentration of 80.6 mg·L^−1^. However, when the ozone concentration was increased from 60 mg·L^−1^ to 80 mg·L^−1^, the COD removal only increased very little. The COD decreased with increasing ozone concentration. During the initial degradation period, the degradation rate was the highest at both low and high ozone concentrations. The degradation rate decreased with reaction time. The rate at a low ozone concentration decreased more significantly than at high ozone concentration. Although high ozone concentration is desirable for COD removal and degradation rate, the utilization efficiency of ozone decreased with increasing ozone concentration. The ozone utilization efficiency of the five-reactor device was three times higher than that of three tandem reactors, demonstrating that ozonation utilization efficiency can be improved by increasing the number of tandem reactors. Ozonation in tandem reactors is a promising approach for PML treatment.

## 1. Introduction

Polymerization mother liquid (PML) is one of the main sources of wastewater in the production of polyvinyl chloride (PVC), which is currently produced mainly through a suspension polymerization process using vinyl chloride monomer (VCM) in the chlor-alkali industry [1]. The main characteristics of PML are [2] (1) Very large volume. The production of one ton of PVC results in producing 3–4 tons of PML. (2) High turbidity. The mass concentration of the suspended solids is 30–50 mg·L^−1^; these solids consist mainly of PVC granules. (3) Low concentration of refractory organic pollutants, with typical chemical oxygen demand (COD) values of 100–400 mg·L^−1^. (4) Poor biodegradability. The BOD_5_/COD values of the PML range from about 0.2 to 0.3. It contains additives such as initiators, and terminators which are added during the polymerization process. These additives contain small amounts of organic matter, but many of them are toxic and difficult to degrade. Possible organic compounds in wastewater are: 1) The initiator: cumyl peroxyneodecanoate, *tert*-butyl peroxyneodecanoate, peroxide dicarbonate-2 (ethyl hexyl) ester, xanthan gum, fatty alcohol polyoxyethylene ether; 2) dispersing agent: polyvinyl alcohol (PVA); 3) antifoaming agent: polyether (glycerol polyoxyethylene polyoxypropylene ether), ethylene glycol, Tween 80 (polyoxyethylene sorbitan oleate), methanol, polysiloxane SPJ-201 non-ionic defoaming agent, Twain 21 (polyethylene glycol laurate sorbitol), non-ionic glycerol polyoxypropylene ether, polyoxyethylene, pPolyoxypropylene block polymer; 4) terminating agent: epoxidized soybean oil, 2,6-2-*tert*-butyl-4-methylphenol (BHT), isocaprylic acid, zinc isoocatanoate, diethylhexyl phthalate, α-methylstyrene, acetone thiosemicarbazone (ATSC), 12% methanol, bisphenol A. 5 antioxidants: 3-(3, 5-di-*tert*-butyl-4-hydroxyphenyl) *n*-octadecanol propionate (antioxidant 1076), cyclododecane, 2-pentadecane acrylate, lauryl acrylate, 3-sulfhydrate dodecyl propionate, etc. Many of these organic auxiliaries are toxic and difficult to degrade and tend to remain in the PML after polymerization. Polyvinyl alcohol (PVA) used as a dispersant that is very difficult to degrade. The tiny PVC particles are suspended in the water, and 85% of the PVA absorbed on the surface of the PVC particles is released into the polymerization mother fluid. PVA usually exists in a colloidal state in water and can form a large amount of foam and promote the migration of heavy metals [2]. Thus, the development of easy-to-operate, highly efficient, and economical process for PML treatment is practically important.

Traditional treatments for PML include coagulation/flocculation [3], filtration, and biotreatment. Additional technologies such as membrane separation have emerged [4,5,6]. Coagulation/flocculation or filtration can effectively remove suspended solids but cannot remove dissolved pollutants effectively. Filtration is limited by its low speed, high power consumption, and the need to treat the used filters. The efficiency of bio-treatment of PML is usually low, because PML is relatively chemically inert and contains many toxic and refractory substances that can inhibit the growth of microorganisms and reduce the ability of biochemical processes, resulting in a low speed of treatment [7,8,9]. The membranes can easily become clogged by residual PVA or algae, and therefore require periodic reverse cleaning and replacement, increasing the cost of this already-expensive treatment method. Most PVC manufacturers in China usually currently utilize secondary bio-treatment facilities combining with membrane technologies for the advanced treatment and reuse of wastewater. Theoretically, it would be desirable to pre-treat the PML with advanced oxidation technique capable of improving the biodegradability and breaking down the polymer chains of PVA and toxic and refractory substances, and then processing the pretreated PML with biotreatment facilities and/or membrane technologies. Along these lines, in the work reported here, the ozonation efficiency of the tandem reactors for the degradation of PML, along with the effect of ozone concentration, the number of reactors utilized in series, and the reaction time on the chemical oxygen demand (COD) removal were investigated.

Advanced oxidation processes (AOPs) utilizing highly reactive oxidizing radicals have been widely employed and have shown excellent performance in the degradation of persistent organics [9,10,11,12,13]. Over the last decade, ozonation has become one of the most effective AOP processes for the removal of organic pollutants, due to its ability to both decrease toxicity and increase biodegradability [14,15,16]. The rapid development of the ozone oxidation method in effluent treatment is attributed to its powerful oxidizing capability, high reaction speed, flexible operation, and without generating secondary pollution and sludge. In addition, it is highly efficient in the degradation of organic contaminants, such as pharmaceuticals and personal care by-products, and in the mineralization of toxic and refractory compounds. [9,17,18,19,20,21,22]. The development of ozone generation technology has reduced the cost of ozonation, and ozonation is considered a competitive technique for the advanced treatment for wastewater.

To the best of our knowledge, few studies have evaluated the degradation of PML using the ozonation with tandem reactors to improve the efficiency of ozone utilization. This study focused on assessing the effective degradation of the PML using devices with three or five tandem reactors, which were designed to estimate the effectiveness and significance of cascade ozone utilization on improving ozonation efficiency. The molecular degradation mechanism, mass transfer process, the effect of increasing the number of reactors on the total efficiency of the ozone oxidation reaction, as well as the relationships between the number of reactors, the ozone concentration, and the reaction time with the ozone utilization, were analyzed. The characteristics and concentration gradients in the tandem reactor were analyzed in terms of COD reduction value and ozone concentration change, and the ozone utilization of the three and five tandem reactors were compared and discussed.

## 2. Results and Discussion

### 2.1. Parameters Influencing COD Degradation

The widely accepted mechanism of ozonation involves two reaction pathways, the direct and indirect reaction routes. In the direct reaction, molecular ozone reacts with organic contaminants, whereas in the indirect reaction, organic pollutants are oxidized by highly reactive free radicals derived from the decomposition of molecular ozone [23,24,25]. Some catalytic ozonation processes (COPs) have been reported on single ozonation processes (SOPs) [26]. COP was proposed to be more effectively convert ozone into free radicals [27,28]. However, separation and recovery of the catalyst, which generates secondary pollution and reduces efficiency, have impeded the practical application of COP for wastewater treatment [10]. 

Direct ozonation can be effective when the concentration of the target species which can react quickly with molecular ozone is appropriate [29,30,31,32]. Hautaniemi [33] reported that hydroxyl radical reactions did not contribute to the oxidation of chlorophenol under alkaline conditions. Hong and Zeng [34] proposed that the degradation of pentachlorophenol was initiated via direct nucleophilic attack by ozone as the predominant pathway, while both ozone and secondary hydroxyl radicals were likely to be involved in the subsequent degradation of intermediates. 

It is well established that developing methods to improve ozone utilization efficiency is key to ozone oxidation technology [35]. From engineering perspective, ozone utilization efficiency can be improved by optimizing the operation mode and reactor structure to facilitate the mass transfer of oxidation process [36]. Because of the low solubility of ozone in aqueous solution and its tendency to quickly decompose [37], only a small amount of gaseous ozone molecules entering the liquid phase are associated with the oxidation. Thus, investigation on improving the utilization of ozone has become a key issue for engineering applications.

It is often difficult to determine the composition and concentration of the individual components of a PML, so comprehensive index such as chemical oxygen demand (COD) and total organic carbon (TOC) are often used to characterize the wastewater. These parameters, especially COD, can provide a useful estimation of the ozone oxidation activity in a PML.

In ozonation techniques, the concentration of ozone, effluent pH value, reaction time, and reaction temperature also could influence the COD value, thus directly affect the oxidizing efficiency. Therefore, the above factors were selected as the parameters to be measured in order to assess the efficiency of ozone utilization on the PML.

Usually, the temperature of the reaction affects the reaction rate. However, temperature has contradictory effects in ozonation: on one hand, higher reaction temperature can increase the reaction rate of oxidants with pollutants [38]. On the other hand, the solubility of ozone in water decreases as the temperature increases [39]. Therefore, a suitable reaction temperature is essential for ozonation. According to many researchers [38,40,41,42], 25 °C is the optimal reaction temperature. In practical engineering applications, adjusting the temperature of the PML is difficult; therefore, in this work, the ambient temperature (23 ± 2 °C) was chosen as the reaction temperature of the treated water.

The pH value of the solution is a crucial in ozone oxidation systems. The ionization state of organic molecules and the process of ozone self-decomposition to form free radicals are affected by the pH to varying degrees. The presence of hydroxide ions in water can induce the formation of hydroxyl radicals (·OH) by ozone and improve the efficiency of the ozone oxidation of pollutants [43]. However, when the pH of the solution reaches 9, the half-life of O_3_ is significantly reduced, and its self-decomposition takes only a few minutes [44]. pH values between 8.5 and 9 are more appropriate. This was very close to the pH value of the PML used in the experiment, so there was no need for extensive pretreatment to adjust the pH of the raw PML. The study focused on the ozone concentration, reaction time, and number of reactors on the PML degradation and the effect of ozone utilization.

**Figure 1:**
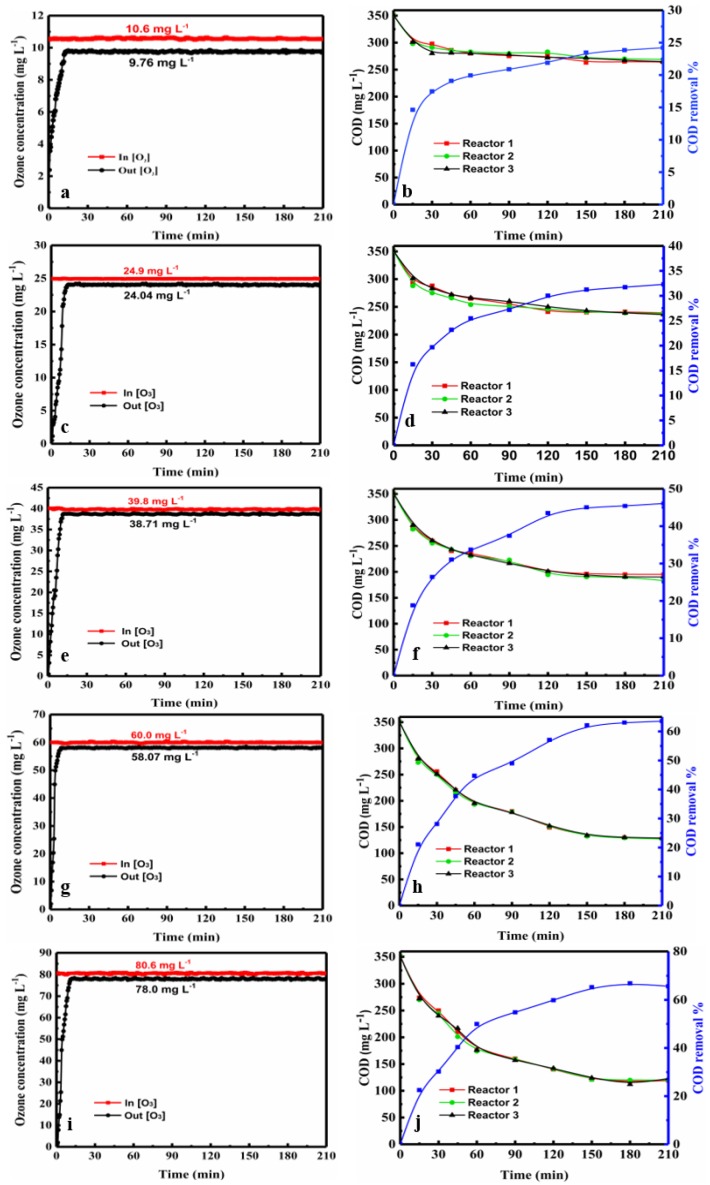
Ozone oxidation in the three tandem reactors. (**a**,**c**,**e**,**g**,**i**) show the variation in the ozone concentration at the inlet and outlet over time for inlet concentrations of 10.6, 24.9, 39.8, 60.0, and 80.6 mg·L^−1^, and (**b**,**d**,**f**,**h**,**j**) show the change in the COD in each of the three reactors during the ozonation process over time.

**Figure 2:**
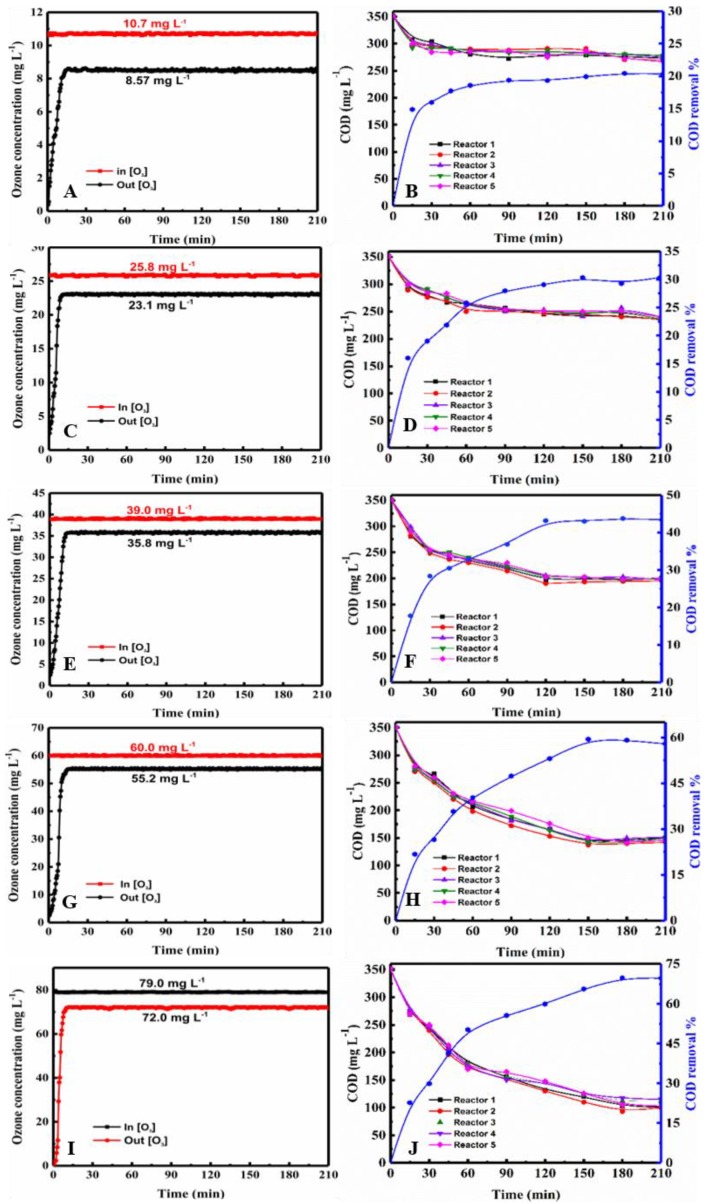
Ozone oxidation in the five tandem reactors. (**A**,**C**,**E**,**G**,**I**) show the variation in the ozone concentration at the inlet and outlet over time for inlet concentrations of 10.7, 25.8, 39.0, 60.0, and 70.0 mg·L^−1^, and (**B**,**D**,**F**,**H**,**J**) show the change in the COD in each of the three reactors during the ozonation process over time.

The PML degradation experiments were carried out at ozone concentrations range of 10, 25, 40, 60, and 80 mg·L^−1^. And the COD was measured at various time intervals in each reactor. The ozone concentrations at the inlet and outlet of the reactors were measured online by the ozone detector every 10 s during the reaction time of 210 min. The data for the three tandem reactors and five tandem reactors are shown in Figure 1 and Figure 2 (data are also shown in Tables S1–S10), respectively. 

As shown in Figure 1 and Figure 2, average inlet ozone concentrations in both devices were virtually identical. In both figures, the COD value decreased with increasing ozone concentration over time. The COD value decreased rapidly at early stage, and then declined. At a given ozone concentration, the COD curves of each reactor are clustered together very closely in both three and five tandem reactors, indicating that the degradation behavior in the reactors are similar. The plots of COD vs. time were close together and very similar for both three and five tandem reactors, indicating that the number of reactors had little impact on the COD decrease (Figures S1 and S2). With the ozone concentration at the inlet was increased, the difference in the concentrations at the inlet and outlet of the reaction device was increased. Moreover, the ozone concentration differences of the five tandem reactors were greater than that of three tandem reactors. This indicated that as the number of tandem reactors increased, a concentration gradient began to appear.

As discussed above the curves of COD over time at given ozone concentration were very similar in both the three and five tandem reactors. This indicates that the number of reactors had little effect on the COD. Thus, the effects of ozone concentration and reaction time on the COD were investigated.

In this work, COD removal of the ozonation system was calculated using Equation (1):

(1) $$\mathrm{COD}\;\mathrm{removal}\;\%\;=\;\frac{{\mathrm{COD}}_{\mathrm{initial}}{-\mathrm{COD}}_{\mathrm{ozonated}}}{{\mathrm{COD}}_{\mathrm{initial}}}$$

#### 2.1.1. Effect of Ozone Concentration on COD 

As shown in Figure 1 and Figure 2, the COD curves over time of each reactor were very similar. The ozone concentrations at the inlet and outlet over time were 10.7, 25.8, 38.9, 60.0, and 79.0 mg L^−1^ respectively. B, D, F, H, J depicts the change in COD in each of the five reactors during the ozonation process. Note: A and B, C and D, E and F, and G and I represent the same conditions.

**Figure 3:**
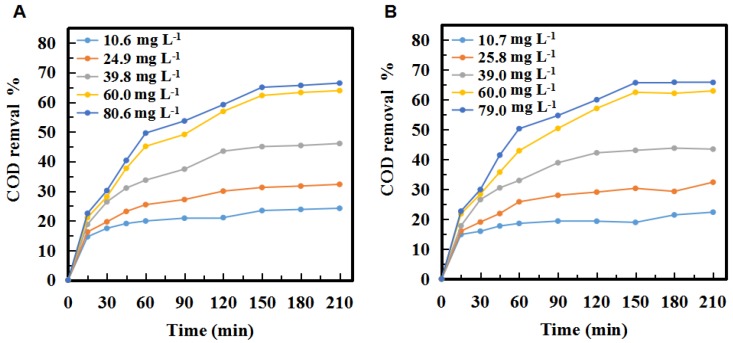
Changes in the COD removal % in the second stage of the (**A**) three tandem reactors and (**B**) five tandem reactors over time at different ozone concentrations.

The ozone concentration and reaction time on COD in three tandem reactors and five tandem reactors were shown in Figure 3. As can be seen, the trends of COD removal of the second stage in the three and five tandem reactors were very similar. In both cases, COD removal increased with the increase of ozone concentration over time. Figure 3 showed that the COD removal of the PML was significantly increased with increasing ozone concentration. The COD removal percentages were up to 66.4% and 65.7% when the concentration of ozone reached 80.6 mg·L^−1^ and 79.0 mg·L^−1^ in the three- tandem reactors and five tandem reactors, respectively, at 210 min. 

The COD removal increased by 8.1% when the ozone concentration was increased from 10.6 mg·L^−1^ to 24.9 mg·L^−1^ in the three tandem reactors (Figure 3A). It was 13.8% and 17.8% when the ozone concentration was increased from 24.9 mg·L^−1^ to 39.8 mg·L^−1^ and from 39.8 mg·L^−1^ to 60.0 mg·L^−1^, respectively. However, with the ozone concentration increased from 60.0 mg·L^−1^ to 80.6 mg·L^−1^, the COD removal only increased by 2.5%.

**Figure 4:**
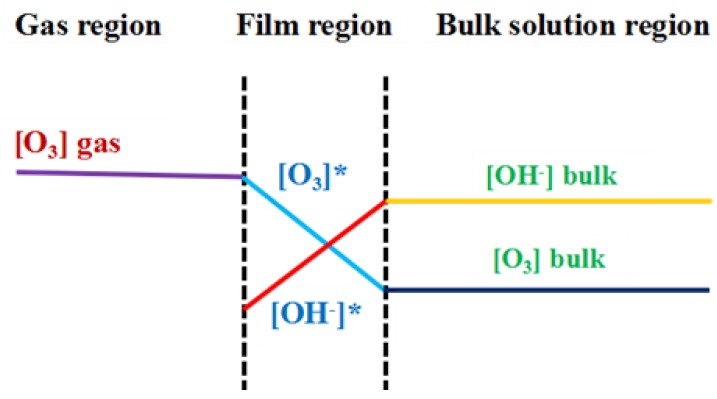
The two-film model for ozone oxidation process [47,48]. [O_3_] gas: the concentration of ozone in the gas region; [O_3_]*: the concentration of ozone in the film region; [O_3_] bulk: the concentration of ozone in the bulk region; [OH^−^]*: the concentration of OH^−^ in the film region; [OH^−^] bulk: the concentration of OH^−^ in the bulk region.

**Figure 5:**
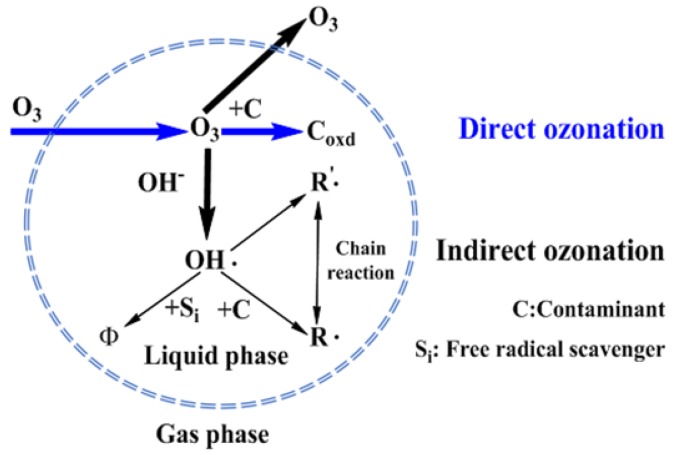
The two types of reactions of ozone in aqueous solution [49]. The blue dashed border represents the phase interface between the gas and liquid. According to the two-film model theory, the two dashed blue lines represent the gas-liquid film resistance, as shown in Figure 4. The area outside the circle represents the gas phase, and the ozone dissolves into the liquid phase.

In summary, the COD removal gradually increased and then reached saturation. The trend of the COD removal in the five tandem reactors was similar to that of the three tandem reactors (Figure 3B). Theoretically a higher ozone concentration would increase the probability of ozone contacting with pollutants, and then increase the degradation efficiency. However, in fact, when the ozone concentration was increased to 80 mg·L^−1^, the COD value decrease only slightly, indicating there was no need to increase the concentration after it reached a certain level, as it did not significantly increase the degradation of the PML. There are three possible explanations for this. First, the reaction of ozone in PML is a heterogeneous, continuous, and competitive gas-liquid reaction. Only when ozone molecules enter the solution from the gas region according to the two-film model (Figure 4) can they react with the organic pollutants. The maximum solubility of ozone in water is fixed. Once the consumption and supply reach a balance, increasing the ozone concentration further is not necessary. Secondly, ozonation is a complicated process that involves direct and indirect reaction pathways (Figure 5). In the classic Criegee mechanism [45], ozone molecules directly react with organic pollutants. These may generate additional aldehydes, saturated carboxylic acids, ketones, etc. The ozone dissolved in the water is unstable, and will quickly decompose to form free radicals, especially hydroxyl radicals. The indirect reaction of ozone, which produces hydroxyl radicals, begins to compete with the direct reaction by molecular ozone. As the ozone concentration is increased, the production rate of intermediates such as small molecular acids will increase, and the pH of the solution will decline rapidly, thus affecting the self-decomposition of ozone [15]. Thirdly, excess ozone can react with hydroxyl radicals [14,46] and free radicals, which could affect the degradation of organic pollutants in the PML. Excess ozone consumption also consumes more electricity, increasing the cost of the oxidation process.

Figure 3 also showed the curve of the COD change over time at various ozone concentrations. The COD decreased very rapidly within the first 15 min. According to the two-film theory as shown in Figure 4, the gas flow diffusion rate is proportional to the gradient in the ozone concentration of the gas phase. The higher the ozone concentration in the gas phase, the stronger mass transfer force will be, and more gas phase ozone molecules that can pass through the air film. However, when the ozone concentration increased to certain level, the excessive amount of ozone is unable to have efficient mass transfer and decomposes into oxygen molecules or directly overflows. So the exit gas contains significant residual ozone gas. As the ozone concentration continues to increase, the curve of the percentage COD removal flattens out, and the excessive amount of ozone actually negatively impacts on the efficiency of the ozone.

The reaction process between ozone and the organic pollutants is a series of diffusion and reaction. The process of ozone diffusion involves the gas-liquid interface resistance and the chemical reaction process after ozone enters the liquid. The COD decreasing rate was very fast at the beginning. The ozone diffusion rate at the beginning was much faster than that of the late stage. Initially, there were no ozone molecules in the solution. When the ozone broke through the gas and liquid films and entered the liquid phase, the concentration gradient between the gas and liquid phases was very large, resulting in a high ozone diffusion rate. This was attributed to the rapidly increasing ozone concentration in the reactors at the beginning of the reaction. The rate of ozone degradation of the pollutants depends on the oxidation characteristics of the ozone itself and the concentration of ozone in the liquid phase. It is also related to the structure and concentration of the pollutants that react with ozone. Pollutants can be divided into those that are easy to be degraded by ozone and those that are difficult to be degraded. Ozone oxidation is partial oxidative degradation of organic pollutants rather than complete mineralization. The easily degradable organics were oxidized completely at the beginning in the PML. Figure 3 showed that in the first 15 min of the reaction, the reaction rate was very fast whether the ozone concentration was high or low. The curve of the COD removal percentage for a high concentration of ozone (60 mg·L^−1^) required 150 min to reach a plateau, indicating the presence of small amounts of difficult-to-degrade substances, some of which begin to degrade with increasing reaction time at high ozone concentration.

The slope of the lines in Figure 3 showed that the COD decreased very rapidly during the initial ~15 min of ozonation, then gradually leveled off. The COD decrease rate increased significantly as ozone concentration was increased from 10 mg·L^−1^ to 40 mg·L^−^^1^. However, the increase in the COD decrease rate was smaller with increasing ozone concentration from 40 mg·L^−1^ to 60 mg·L^−1^, and increasing it further from 60 mg·L^−1^ to 80 mg·L^−1^ had a nearly negligible effect. This trend could be attributed to the ozone dissolution equilibrium as was discussed before. Above certain concentration level, the ozone concentration within the PML did not increase, and thus the COD decrease rate was not improved. Additionally, results showed that the number of reactors used had little effect on the degradation rate. The same trend was observed for both the three and five tandem reactors. 

#### 2.1.2. Effect of Ozonation Time

As can be seen from Figure 3, COD decreased with increasing reaction time, and there is a critical point that divides the entire curve into two distinct time periods: the fast-reaction stage and the slow-reaction stage. The first stage occurred within 15 min at the beginning of the ozonation, indicating rapid COD reduction and a very high COD removal rate. Subsequently, the decreasing slope increased with increasing ozone concentration. About 80% of the total COD reduction occurred during this short period. This could be ascribed to the high ozone oxidation kinetics and the high concentration of the components as well as their reactivity with ozone in PML. The rate of COD decomposition was very rapidly, presumably due to the relatively low organic matter concentration (low COD value).

In general, when the concentration of pollutants is high at the beginning of the reaction, rapid kinetics and direct ozone reaction will occur. In the first stage, ozone oxidation occurred rapidly and the pollutants in PML were mainly removed by direct ozone reaction [49]. Another stage was observed between 15–210 min of treatment. In this stage, the decreasing rate of COD was reduced significantly, and the curve became flat with reaction time. This indicates that the oxidative degradation approach the low-rate stage, i.e., a low-rate dynamic system. In this stage, the indirect reaction pathway, which involves free radical oxidation, competes to consume ozone to remove the pollutants as soon as ozone is present in PML. The indirect ozone reaction plays a major role in the low-rate system with increasing time. The indirect reaction could be triggered by the reaction of ozone with hydroxide ions, which are the first step of the chain reaction and the rate-limiting step that determines the reaction rate. After 60 min of ozonation as shown in Figure 3, the change rate of COD removal after 60 min (that is, the slope of the curve) was slower than the initial degradation rate, the compounds that were difficult to be oxidized in water were left and accumulated, and the ozonation system changed to a medium-rate or low-rate dynamic system [50]. 

#### 2.1.3. Effect of the Number of Reactors 

**Figure 6:**
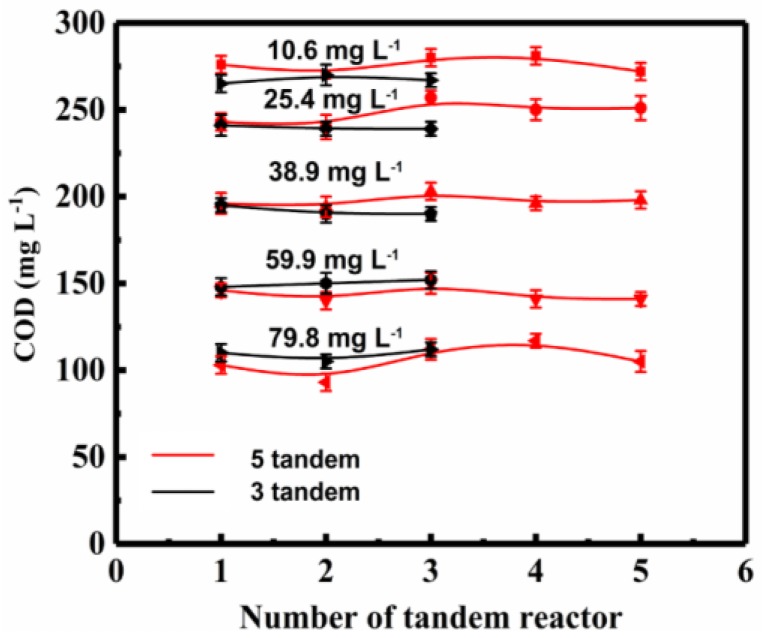
The COD at different ozone concentrations at 180 min in the three and five tandem reactors.

In order to evaluate the influence of number of tandem reactors on COD, the COD value in each reactor was measured, and the differences between the three-tandem reactor and five tandem reactors were compared (Figure 6). 

The changes of COD value among the same reactors in the three tandem reactors and five tandem reactors were very small. Although the fluctuations among the five tandem reactors were about the same as that in three tandem reactors, the changes in the COD values of the five-tandem reactors device were greater than that of the three tandem reactors. This indicated that the concentration gradient of ozone was more obvious at the beginning of the reaction for the five tandem reactors, which was also consistent with the variation of the concentration difference between the inlet and outlet.

When comparing the COD value decrease of the three-tandem reactors and five-tandem reactors, it was observed that the COD decrease rate was similar, except that the five-tandem reactors showed a relatively obvious ozone concentration gradient between the reactors. Increasing the number of tandem reactors from three to five increased the wastewater treatment capacity without costing extra energy. In the experiment, the concentration of ozone did not decline significantly between reactors, implying the amount of ozone reacted in the first reactor was small. In addition to the low solubility of ozone, its half-life is relatively long, and thus each subsequent reactors had only slightly decreased ozone concentration. When the process scaled up to industrial reactors, the cascade effect of ozone concentration would be more obvious, and the COD value change would vary more significantly between the reactors in tandem. Results also implied that at appropriate conditions, wastewater treatment capacity could be raised by increasing the number of tandem reactors. However, one should keep in mind that with the increase in the number of tandem reactors, the flow resistance would also increase accordingly. In our experiments, indeed we observed that when the number of reactors increased to six, the resistance increased significantly, and the turbulence in each reactor and mass transfer effect decreased significantly. Increasing the number of tandem reactors also increased the complexity of overall process operation.

#### 2.1.4. PVA Degradation Pathway and Mechanism

PML usually contains PVA, residual vinyl chloride monomer, and small amounts of other additives. However, many of them are toxic (biphenyl A, hydroquinone, methanol, etc). It also contains some hydrocarbons and carboxylic acids [26]. HO· can decompose most of these substrates efficiently, but not all of them [51].

As the major refractory polymeric pollutant in the PML, the degradation of PVA had a strong positive correlation with the reduction of COD in PML. Therefore, the analysis of the degradation mechanism and pathway of PVA is an important for PML treatment. As strong oxidant, the ·OH radical can oxidize organic compounds fairly quickly by destroying chemical bonds such as C–H, C–C, C–O, and C=C bonds [51]. Like other organic compounds, PVA in wastewater can be broken down into smaller pieces (radicals, represented as R·) when attacked by ·OH. Further chain scission can occur to the R· radicals until they are decomposed completely into CO_2_ and water. Some ketones/enols could be formed during the process of mineralization due to the presence of ·OH and O_2_ [51,52]. 

**Figure 7:**
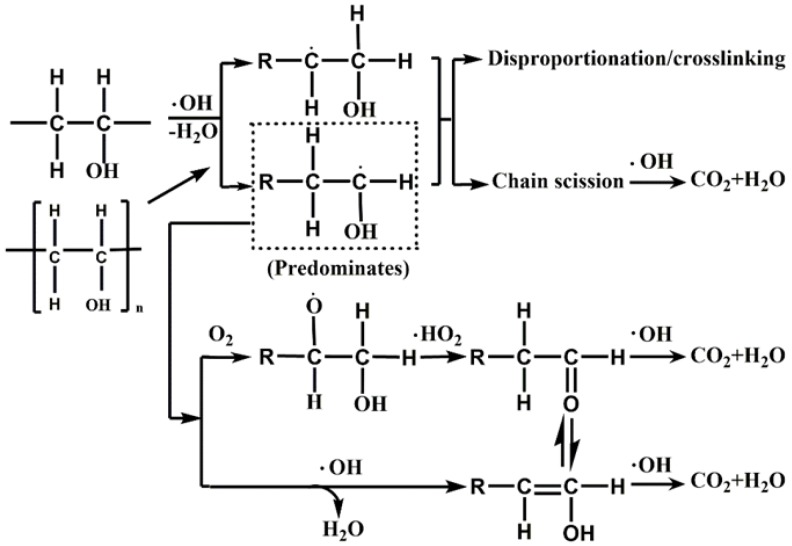
Possible pathways of PVA degradation in the presence of ·OH and O_2_ [53].

Some possible pathways for the radical-induced degradation of PVA in wastewater are shown in Figure 7. In the first step, the high-molecular-weight contaminant PVA is activated by ·OH or H·. These radicals can attack at either α or β position to induce dehydration or dehydrogenation to produce a small molecule containing R·. The small molecule then undergo reaction with O_2_ or ·OH to form a ketone or enol, which then could be further oxidized to a carboxylic acid and eventually mineralized [52]. In addition to this mineralization pathway, small molecules containing R· may undergo disproportionation or cross-linking reactions with each other; the resulting products will then be further broken down by the action of ·OH until total conversion of H_2_O and CO_2_ is achieved [51,53].

Based on the above mechanism, at the initial stage of the reaction, the amount of HO· produced was insufficient to destroy all the PVA polymer chains. Therefore, its removal efficiency was low, and the COD removal was low. With increasing ozone input, the oxidative degradation of the large molecule pollutant PVA in the wastewater start to occur, the removal efficiency of PVA will continuously increase, and the COD removal will be increased. However, once a certain O_3_ concentration is reached, the resulting free radicals are consumed by both oxidative reaction and quenching one another [54]. Therefore, the removal of PVA will gradually level off. Although HO· can destroy the long molecular chains of PVA, many intermediate products such as acetone and oxalic acid may still exist in the wastewater [55], as they could not be oxidize further with HO·.

### 2.2. Analysis of Ozone Utilization

Ozone utilization efficiency is the key issue for industrial wastewater treatment. The poor efficiency in ozonation technologies has drawn considerable concern [29,30,31]. The reaction of ozone in wastewater is a heterogeneous continuous and competitive gas-liquid reaction. That is, ozone is transported from the gas phase (oxygen or air) to the liquid phase, and diffusion occurs at the same time as reaction with the pollutants in the water. In addition, the solubility of ozone in water dictates the concentration distribution of ozone molecules in between the two phases. The reaction of ozone with organic and inorganic substances in aqueous solution generally go through the direct reaction of molecular ozone or through indirect radical reactions. This involves the reaction of the hydroxyl radical generated by the decomposition of ozone in water, as shown in Figure 5. In this multi-step reaction process, the dissolution of ozone into the liquid phase is the controlling step, as ozone in the liquid is either directly involved in molecular reactions, generates hydroxyl free radicals to start the indirect reaction, or escapes back into the gas phase. Additionally, many factors that cause ozone to decompose and limit its solubility in water will affect ozone oxidation. The amount of ozone that can actually participate in the degradation of organic pollutants is very limited, and thus the ozone utilization efficiency is quite limited. Such low ozone efficiency greatly limits its real application for industrial wastewater. 

In this work, the mass balance of ozone in the liquid and gas phases shown in Equation (2) was applied [56].

(2) $$[O_{3}{]}_{T}\;=\;[O_{3}{]}_{C}\;+\;[O_{3}{]}_{R}\;+\;[O_{3}{]}_{O}$$

where [O_3_] _T_, [O_3_] _C_, [O_3_] _R_, and [O_3_] _O_ represent the total used, concentrations of consumed, residual, and off-gassed ozone, respectively.

The percentage of ozone consumption was measured as the ratio of the sum of the consumed ozone [O_3_] _C_ and residual ozone [O_3_] _R_ to the total used ozone, as described in the following equation:

(3) $$\mathrm{Ozone}\;\mathrm{consumption}\;\mathrm{ratio}\%\;=\;\frac{[O_{3}{]}_{T}-[O_{3}{]}_{O}}{{\left[O_{3}\right]}_{T}}$$

#### 2.2.1. Change in the Ozone Concentration in the Three-Tandem Reactors and Five-Tandem Reactors

The change in the ozone concentrations at the inlet and outlet of the three-tandem reactors and five-tandem reactors over time is shown in Figure 1a,c,e,g,i and Figure 2A,C,E,G,I. As discussed previously, the difference in ozone concentration at the inlet and outlet of the three-tandem reactors and five-tandem reactors gradually increased with increasing ozone concentration. That is, the higher the ozone concentration at the inlet, the more obvious the change in the ozone concentration at the outlet. The higher ozone concentration could increase the mass transfer force of the gas and liquid phase, increasing the diffusion rate. This indicated that as the concentration of the gas phase increased, more ozone would enter into the liquid phase to participate in the oxidative reaction. The change in ozone concentration between the inlet and outlet was indeed affected by the number of reactors. A comparison of the difference in the ozone concentrations of the three-tandem reactors and five-tandem reactors is shown in Figure S4.

#### 2.2.2. Comparison the Ozone Consumption Ratio of the Three-Tandem Reactors and Five-Tandem Reactors

Table 1 presents the inlet and outlet ozone concentration, difference in ozone concentration at the inlet and outlet, and the consumption rate of ozone of the three-tandem reactors and five-tandem reactors. The difference in ozone concentration was calculated by subtracting the outlet concentration from the inlet ozone concentration. The ozone consumption was calculated as (inlet ozone concentration-outlet ozone concentration)/inlet ozone concentration according to Equation (3).

In both cases, the consumption of ozone decreased with increasing ozone concentration. Thus, increasing the number of tandem reactors improved the ozone utilization efficiency, while increasing the inlet ozone concentration did not. Interestingly, the ozone consumption ratio was highest at low ozone concentrations. This is contrary to the observation that the COD value decreased with increasing ozone concentration. Although the ozone consumption increased with the number of reactors, the pressure required for normal operation of the continuous reaction must be provide. Thus, there is a trade-off and a balance between increased COD reduction value and decreased ozone consumption when choosing the ozone concentration and number of reactors for the PML ozonation process.

#### 2.2.3. Ozone Utilization of the Three-Tandem Reactors and Five-Tandem Reactors

**Figure 8:**
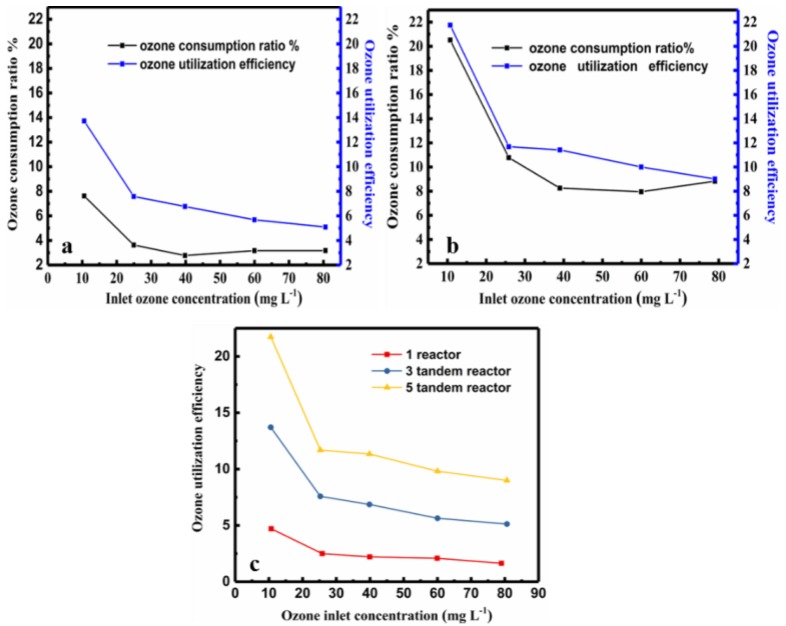
Comparison of the ozone consumption ratio and ozone utilization efficiency at different inlet ozone concentrations at 180 min. (**a**) Three-tandem reactors (**b**) five-tandem reactors (**c**) The relationship between the utilization efficiency % and the inlet ozone concentration for the three- tandem reactors and five-tandem reactors at 180 min. The red line in (**c**) is only one reactor for comparison.

To optimize the ozone utilization with tandem reactors, several important factors must be considered: the inlet ozone concentration, number of reactors, amount of reduction of COD, and reaction time. In our experiment, we define the ozone utilization at 180 min using the following expressions:

(4) $$m\left(O_{3}\right)=\int_{0}^{180}{\left[O_{3}\;\right]}_{T}\;Q\;\mathrm{dt}\;=\overset{180}{\underset{0}{\sum }}{\left[O_{3}\right]}_{n}Q\;\Delta t\;(\Delta t=0.5\;\min)$$

(5) $$\mathrm{ozone}\;\mathrm{utilization}\;\mathrm{efficiency}\;=\;\frac{\left({\mathrm{COD}\;}_{\mathrm{initial}}{\;-\;\mathrm{COD}\;}_{\mathrm{ozonated}}\right)\;}{m\left(O_{3}\right)}=\frac{\sum \;\left(\Delta \mathrm{COD}\;\mathrm{of}\;\mathrm{all}\;\mathrm{reactors}\right)}{m\left(O_{3}\right)}$$

where Q (L·min^−1^), is volume flow of ozone, [O_3_] _T_ (mg·L^−1^), represent the total ozone used [29,57]. Equations (4) and (5) is used in the tandem reactors. The calculated results in Figure 8 provide a direct correlation between ozone utilization efficiency and the inlet ozone concentration. As shown in the Figure 8c, the ozone utilization of the five-tandem reactors was significantly higher than that of the three-tandem reactors. Additionally, the ozone utilization decreased with increasing ozone concentration, similar to the ozone consumption.

The ozone utilization efficiency and the ozone consumption show the similar trends with ozone concentration in the process of ozonation degradation of wastewater, as shown in Figure 8. 

The ozone consumption calculation involves three parts as shown in Equation (2): the ozone in the gas, in the liquid phase, and in the reaction, ∆ [O_3_] = [O_3_] _C_+ [O_3_] _R_ = [O_3_] _T_ − [O_3_] _O_, while the utilization efficiency reflects the reaction part. In fact, the consumed ozone includes two parts. The first part is the ozone that has been reacted, i. e. the ozone that is actually reacts with pollutants to decrease the COD. The second part is the residual ozone, which has not participated in the reaction; this includes the ozone that dissolved in the water, escaped to the air, decomposed itself; etc. The utilization efficiency reflects the only the reaction part, i.e., the ozone that reduced the COD value.

When the two lines are closer, it indicates that more ozone was consumed in the reaction, that the utilization efficiency was improved. The ozone utilization efficiency is a more accurate index to reveal how much ozone is used in for the decrease of COD. The two lines are obviously closer in the plot of the five-tandem reactors than that of the three-tandem reactors (compare Figure 8a,b). As shown in Figure 8c, the ozone utilization efficiency of the five-tandem reactors was two times higher than that of three-tandem reactors. Thus, increasing the number of reactors can effectively improve the ozone utilization efficiency. However, the COD decrease rate and ozone utilization efficiency showed contradictory and were found to have non-linear relationships with ozone concentration, reaction time, and number of tandem reactors as the variables. The optimal reaction parameters depend on not only the target of the oxidative reaction, but also the concentration of the wastewater. If the shortest reaction time is the objective, the use of a high ozone concentration and more reactors is desirable. However, if the objective is to have high ozone utilization efficiency, increasing the ozone concentration may not be helpful, as there is a tradeoff between the inlet ozone concentration and the ozone utilization; i.e., a high ozone concentration may result in less ozone utilization efficiency.

### 2.3. Biodegradability Analysis

The advanced oxidation process such as ozonation with tandem reactors could be helpful for pretreatment of major industrial wastewater. It was found that ozone played an important role in combination process of wastewater treatment [47]. Therefore, the ozonation process could improve the biodegradability of wastewater [58], but also improve the sedimentation performance and reduce the sludge yield in some cases [50]. In the work reported here, it was impressive that the biodegradability of PML wastewater got significantly improved with ozonation using tandem reactors. 

The BOD_5_/COD ratio is used to evaluate biodegradability. In general, the greater the BOD_5_/COD ratio, the better the biodegradability. A BOD_5_/COD ratio greater than 0.3, indicating that the wastewater can be degraded by microorganisms. A BOD_5_/COD ratio less than 0.3, indicating that the organic matter in the wastewater is highly toxic and difficult to be degraded. If the BOD_5_/COD ratio is greater than 0.4, the wastewater can be easily degraded. 

After being treated at ozone concentration of 80 mg·L^−1^ for 210 min, the COD of the PML decreased from 351 to 117 mg·L^−^^1^. BOD_5_ was improved from 35 to 47 mg·L^−1^. The results showed that the BOD_5_/COD ratio of PML exceeded 0.4 after the wastewater was oxidized for 210 min at an ozone concentration 80 mg·L^−1^. This result implied that ozonation could effectively improve the biodegradability of PML. The improved biodegradability was ascribed to converting refractory, highly toxic macromolecular organic pollutants into small organic molecules with low (or no) toxicity by ozonation. 

**Figure 9:**
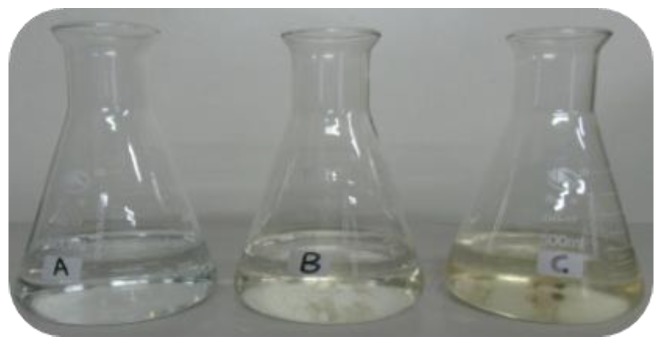
Images of biodegradation of the oxidized PML. (**A**) Raw PML, (**B**) ozonated PML after 14 days at room temperature, and (**C**) ozonated PML after 30 days at room temperature. (Experimental conditions: oxidation time: 210 min, oxidation ozone concentration: 80 mg·L^−1^, room temperature).

The oxidized PML was allowed to stand at room temperature for 30 days. The photo in Figure 9 depicts untreated PML (A), ozonated PML after 14 days at room temperature (B), and ozonated PML after 30 days at room temperature. Nature growing mold was visually observed in ozonated PML after 30 days at room temperature. This result clearly implied that ozonation indeed improved the biodegradability of sample by degrading the toxic and harmful compounds in PML wastewater.

## 3. Materials and Methods 

### 3.1. PML Samples

PML samples were taken from the polymerization section of a large chlor-alkali chemical plant in Xinjiang, China belonging to Zhongtai Chemistry. A typical suspension polymerization technique is employed in the plant for the production of PVC. The main pollutant present in the PML was PVA, with a content of about 1.3–4.8 mg·L^−1^. The COD in the PML from this plant usually ranges from 100 to 400 mg·L^−1^, while its BOD_5_/COD value ranges from about 0.1 to 0.2. The concentration of ammonia nitrogen (NH_3_–N) is about 12 mg·L^−1^, the pH ranges between 8.5 and 9.5, the turbidity is about 5 NTU, and the highest SS is 16 mg·L^−1^. This wastewater is classified as low-concentration refractory wastewater, and the total phosphorus (TP) in the PML is relatively low. The COD of each of the raw PML samples was measured using the methods described later in this section; the initial COD was 351 mg·L^−1^.

### 3.2. Apparatus and Analysis Methods

The chemical oxygen demand (COD) was determined using a COD colorimeter (DR1010, HACH, Loveland, CO, USA) with a digestion device (DRB200, HACH) according to the US EPA approved HACH Method No. 8000. The pH value was measured using a pH meter (pHS-3C, Leici, INESA Scientific Instrument Co., Ltd., Shanghai, China) with Glass electrode method (Chinese National Standard GB 6920-86). NH_3_-N and TP were determined using an ultraviolet/visible spectrophotometer (T6xinyue, HuaBi Scientific Instrument Co., Ltd. Nanjing, China) with Nascar reagent photometry and Chinese National Standard GB 11893-89. PVA was analyzed using the UV–Vis spectrophotometer (DR 6000, HACH) at 690 nm, based on the blue color produced by the reaction of PVA with iodine (analytically pure, Tianjin Zhiyuan Chemical Reagent Co. Ltd., Tianjin, China) in the presence of boric acid (analytically pure, Tianjin Zhiyuan Chemical Reagent Co. Ltd.) [2]. The turbidity of the PML was determined using a turbidity meter (ET93810, Lovibond^®^ Tintometer Group, Dortmund, Germany) according to the US EPA 180.1 method. The BOD_5_ was determined according to the pressure sensor method with an automated measurement apparatus. The measurement of BOD (20 °C, 5 days) was performed using a pressure sensor method with an automated measurement apparatus (BOD Trak II, HACH).

### 3.3. COD Determination

A 2 mL PML sample was placed in a HACH special digestion colorimeter tube (160 mm × 100 mm) and mixed well with a digestion solution for COD (Cat.2125916, Pk/150, Range 20–1500 mg·L^−1^, HACH) and digested at 150 °C for 2 h in a Trak II digestion apparatus (DRB200, HACH). After cooling to room temperature, the vial was transferred into the COD colorimeter (DR1010, HACH) for COD determination. 

### 3.4. Experimental Set-Up and Procedure

**Figure 10:**
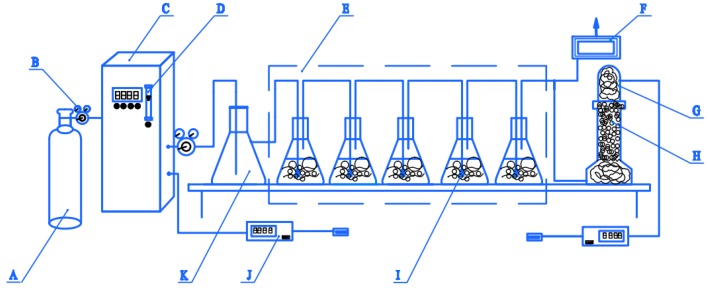
Schematic diagram of the three/five-reactor ozone oxidation system. A: oxygen cylinder; B: pressure reducing valve; C: ozone generator; D: rotor flow meter; E: three- or five-reactor system; F: ozone destruction device; G: drying tower; H: CaCl_2_ particles; I: titanium aerator; J: gaseous ozone concentration detector K: buffer flask.

The ozonation experiment for PML was conducted in the semi-continuous three- or five-reactor system shown in Figure 10, which consisted of three parts. The first part included the ozone generator (NPO/A-100P-S-2, Shandong NIPPON Photoelectricity Equipment Co., Ltd., Jinan, China) with pure oxygen (99.99%, Xinjiang Kangdi Technology Co. Ltd., Urumqi, China) as the feed gas and a gaseous ozone concentration detector (Ideal 2000, Ideal Measurement and Control Technology Co., Ltd., Zibo, Shandong, China). Ozone was generated and delivered to the second part of the three- or five-reactor system, which was composed of one 2 L buffer bottle with a polytetrafluoroethylene tube connected to a rotary flow meter on the ozone generator, which controlled the ozone flow at the outlet. The outlet was connected via a silicone tube to the reactors, which consisted of three or five 500 mL volumetric flasks in series. A titanium alloy ozone diffuser (bore diameter 100 µm) was punched through the rubber plug sealing each bottle and centered at the bottom of each reactor as shown in Figure 10. The third part was the residual ozone detection and treatment system. The outlet for the residual ozone was controlled by a three-way valve. At one branch, the ozone was passed through a drying tower containing CaCl_2_ granules (analytically pure, Tianjin Zhiyuan Chemical Reagent Co. Ltd.) to remove moisture, and then measured by a second ozone concentration detector to determine the residual ozone concentration. The other branch led to an ozone destruction device employed for decomposing of the residual off-gas. The experimental reaction device shown in Figure 10 was airtight. 

The experimental procedure was as follows: 250 mL PML samples were added to the each of the three or five 500 mL volumetric flasks in the device. The flasks were carefully covered with rubber seals to fully close the bottles in sequence, as shown in Figure 10. The oxygen cylinder was opened, the ozone concentration was adjusted to 10.0, 25.0, 40.0, 60.0, or 80.0 mg·L^−1^ by adjusting the pulse width of the ozone generator, and the ozone gas flow rate was set to 10 L·min^−1^. When the ozone flow stabilized and residual gases had been removed from the system, after the concentration of ozone reached the set value and was basically stable, the three-way valve was opened, starting the reaction. Reaction times of 15, 30, 45, 60, 90, 120, 150, 180, and 210 min were studied. After the specified time period, the ozone generator was stopped. After the residual ozone in the reaction system had been removed, the ozone generator switch and the pressure reducing valve of the oxygen cylinder were closed. After allowing the treated PML to rest for the designated stay time, the parameters were determined. All the experiments were repeated three times, and the corresponding mean and standard deviation (SD) of the values were reported as the final data.

## 4. Conclusions

In summary, this study investigated the use of tandem reactors in the semi-continuous operation mode for oxidative degradation of PML wastewater via ozonation. The study focused on improving the ozone utilization efficiency by specifically designed three and five tandem reactors. The ozone utilization efficiency was shown to be a useful comprehensive index for evaluation of degradation of PML wastewater. The removal of COD was fast and effective, with COD decrease up to 66.4%, and the biodegradability of the PML was significantly improved. The rate of COD decrease increased with increasing ozone concentration over time. Increased ozone concentration led to faster reaction while the efficiency of ozone utilization decreased about 60% with the ozone concentration increase from 10 mg·L^−1^ to 80 mg·L^−1^. Advanced oxidation processes utilizing highly reactive oxidizing radicals showed great application potential in the degradation of persistent organics for industrial wastewater. Ozonation coupled with tandem reactors could significantly improve the ozone utilization efficiency and biodegradability of the PML wastewater.

## Figures and Tables

**Table 1:** Ozone concentration (mg·L^−1^) and consumption ratio (%) of the three- and five-reactor devices.

3-Reactor Device				5-Reactor Device			
Inlet O_3_ Conc.	Outlet O_3_ Conc.	ΔO_3_ Conc.	O_3_ Consumption Ratio %	Inlet O_3_ Conc.	Outlet O_3_ Conc.	ΔO_3_ Conc.	O_3_ Consumption Ratio %
10.6	9.76	0.840	7.92	10.7	8.51	2.19	20.5
24.9	24.0	0.900	3.61	25.8	23.1	2.70	10.5
39.8	38.7	1.10	2.76	39.0	35.8	3.20	8.21
60.0	58.1	1.90	3.17	60.0	55.2	4.80	8.00
80.6	78.0	2.60	3.23	79.0	72.0	7.00	8.86

## Supplementary Materials

The following are available online at https://www.mdpi.com/1420-3049/24/24/4436/s1, Table S1: Record and process of the experimental data of COD with time for inlet ozone concentrations of 10.6 mg·L^−1^ in each of the three- reactor tandem ozone reaction devices. Table S2: Record and process of the experimental data of COD with time for inlet ozone concentrations of 24.9 mg·L^−1^ in each of the three- reactor tandem ozone reaction devices. Table S3: Record and process of the experimental data of COD with time for inlet ozone concentrations of 39.8 mg·L^−1^ in each of the three- reactor tandem ozone reaction devices. Table S4: Record and process of the experimental data of COD with time for inlet ozone concentrations of 60.0 mg·L^−1^ in each of the three- reactor tandem ozone reaction devices. Table S5: Record and process of the experimental data of COD with time for inlet ozone concentrations of 80.6 mg·L^−1^ in each of the three- reactor tandem ozone reaction devices. Table S6: Record and process of the experimental data of the change in COD in each of the five reactors during the ozonation process for inlet ozone concentrations of 10.7 mg·L^−1^ in the five- reactor tandem ozone reaction devices. Table S7: Record and process of the experimental data of COD in each of the five reactors during the ozonation process for inlet ozone concentrations of 25.8 mg·L^−1^ in the five- reactor tandem ozone reaction devices. Table S8: F. Record and process of the experimental data of the change in COD in each of the five reactors during the ozonation process for inlet ozone concentrations of 38.9 mg·L^−1^ in the five- reactor tandem ozone reaction devices. Table S9: Record and process of the experimental data of the change in COD in each of the five reactors during the ozonation process for inlet ozone concentrations of 59.9 mg·L^−1^ in the five- reactor tandem ozone reaction devices. Table S10: Record and process of the experimental data of the change in COD in each of the five reactors during the ozonation process for inlet ozone concentrations of 79.0 mg·L^−1^ in the five- reactor tandem ozone reaction devices. Table S11: COD in the second stage of the 3- reactor devices at various ozone concentrations over 210 min. Table S12: COD in the second stage of the 5- reactor devices at various ozone concentrations over 210 min. Table S13: COD removal rate in the second stage of the three tandem reactors change with time at different ozone concentrations. Table S14: COD removal rate in the second stage of the five tandem reactors change with time at different ozone concentrations. Table S15: Calculation of the consumption (%) and Ozone utilization efficiency (%) of the three- reactor tandem devices at 180 min. Table S16: Calculation of the consumption rate (%) and Ozone utilization rate (%) of the five - reactor tandem devices at 180 min. Figure S1: COD in the second stage of the (a) 3- and (b) 5-reactor devices at various ozone concentrations over 210 min. Figure S2: COD in each of the stages of the three- and five-tandem reactors at different levels of ozone concentration at 210 min. Figure S3: The COD at different ozone concentrations in 180 min change with the 3- and 5-tandem reactors. Figure S4: Ozone concentration at the inlet (red color line) and outlet (black color line) of the (a) 3- reactor and (b) 5-reactor tandem devices.
