# Supplementary material for: Improved Ozonation Efficiency for Polymerization Mother Liquid from Polyvinyl Chloride Production Using Tandem Reactors
Source: Molecules. 2019 Dec 4;24(24):4436. doi: 10.3390/molecules24244436 (PMC6943591; doi:10.3390/molecules24244436)
Supplement: Supplementary file 1 [file molecules-24-04436-s001.pdf]

The supporting information

Molecular mechanism and efficiency optimization of ozonation using tandem reactors for polymerization mother liquid from polyvinyl chloride production

Zhiyong Yang [1, 2, 3], Penglei Wang[1, 2], Yagang Zhang *[,1,2,3,4], Xingjie Zan *[,1,2], Wenjuan Zhu [1, 2, 3], Yingfang Jiang [1,2], Letao Zhang[1] and Akram Yasin [1]

[1] Xinjiang Technical Institute of Physics and Chemistry, Chinese Academy of Sciences, Urumqi 830011，China; yangzy@ms.xjb.ac.cn (Z.Y.); wangpl@mail.dlut.edu.cn (P.W.); ygzhang@ms.xjb.ac.cn (Y.Z.); zanxj@ms.xjb.ac.cn (X.Z.); zhuwj@ms.xjb.ac.cn (W.Z.); jiangyf@ms.xjb.ac.cn (Y.J.); zhanglt@ms.xjb.ac.cn (L.Z.); akram@ms.xjb.ac.cn (A.Y.)

[2.] University of Chinese Academy of Sciences, Beijing 100049, China

[3] Department of Chemical and Environmental Engineering, Xinjiang Institute of Engineering, Urumqi
830023, China;

[4] School of Materials and Energy, University of Electronic Science and Technology of China, Chengdu 611731, China

*Correspondence: ygzhang@ms.xjb.ac.cn; zanxj@ms.xjb.ac.cn; Tel.: +86-18129307169
Received: date; Accepted: date; Published: date

Abstract: Polymerization mother liquid (PML) is one of the main sources of wastewater in the chlor-alkali industry. The effective degradation of the PML produced in PVC polymerization using three or five ozone reactors in tandem was designed with focus on improving ozonation efficiency. The ozonation efficiency of the tandem reactors for the degradation of PML, along with the effect of ozone concentration, the number of reactors utilized in series, and the reaction time on the chemical oxygen demand (COD) removal were investigated in detail. The results showed that the COD removal increased as the ozone concentration was increased from 10.6 to 60 mg L^{-1}, achieving 66.4% COD removal at ozone concentration of 80.6 mg L^{-1}. However, when the ozone concentration was increased from 60 mg L^{-1} to 80 mg L^{-1}, the COD removal only increased very little. The COD decreased with increasing ozone concentration. During the initial degradation period, the degradation rate was the highest at both low and high ozone concentrations. The degradation rate decreased with reaction time. The rate at a low ozone concentration decreased more significantly than at high ozone concentration. Although high ozone concentration is desirable for COD removal and degradation rate, the utilization efficiency of ozone actually decreased with increasing ozone concentration. The ozone utilization efficiency of the five-reactor device was three times higher than that of three tandem reactors, demonstrating that ozonation utilization efficiency can be improved by increasing the number of tandem reactors. Ozonation in tandem reactors is a promising approach for PML treatment.

Keywords: polymerization mother liquid, ozonation efficiency, tandem reactor, COD

Table S1. Record and process of the experimental data of COD value with time for inlet ozone concentrations of 10.6 mg L^{-1} in the three- reactor tandem ozone reaction devices.

Time (min)	0	15	30	45	60	90	120	150	180	210
**Reactor 1**	351	300	298	286	280	275	276	263	265	264
**Reactor 2**	351	298	291	285	283	280	283	271	270	269
**Reactor 3**	351	301	280	281	280	278	272	272	267	265

Table S2. Record and process of the experimental data of COD value with time for inlet ozone concentrations of 24.9 mg L^{-1} in the three- reactor tandem ozone reaction devices.

Time (min)	0	15	30	45	60	90	120	150	180	210
**Reactor 1**	351	293	288	271	265	256	241	240	241	239
**Reactor 2**	351	288	275	266	254	251	246	241	239	238
**Reactor 3**	351	301	283	272	266	260	250	243	239	236

Table S3. Record and process of the experimental data of COD with time for inlet ozone concentrations of 39.8 mg L^{-1} in the three- reactor tandem ozone reaction devices.

Time (min)	0	15	30	45	60	90	120	150	180	210
**Reactor 1**	351	283	260	240	236	220	200	196	195	195
**Reactor 2**	351	282	255	243	230	223	194	190	190	183
**Reactor 3**	351	290	260	243	232	216	201	193	190	190

Table S4. Record and process of the experimental data of COD with time for inlet ozone concentrations of 60.0 mg L^{-1} in the three- reactor tandem ozone reaction devices.

Time (min)	0	15	30	45	60	90	120	150	180	210
**Reactor 1**	351	278	256	220	194	180	149	133	130	128
**Reactor 2**	351	273	250	215	193	179	151	132	129	127
**Reactor 3**	351	280	251	221	195	178	152	134	130	129

Table S5. Record and process of the experimental data of COD with time for inlet ozone concentrations of 80.6 mg L^{-1} in the three- reactor tandem ozone reaction devices.

Time (min)	0	15	30	45	60	90	120	150	180	210
**Reactor 1**	351	274	250	210	177	160	140	121	117	119
**Reactor 2**	351	270	245	201	174	159	141	121	120	118
**Reactor 3**	351	272	240	217	176	157	142	124	112	123

Table S6. Record and process of the experimental data of COD in each of the five reactors during the ozonation process for inlet ozone concentrations of 10.7 mg L^{-1} in the five- reactor tandem ozone reaction devices.

Time (min)	0	15	30	45	60	90	120	150	180	210
**Reactor 1**	351	307	304	290	280	272	279	279	276	273
**Reactor 2**	351	296	297	289	290	288	291	291	270	270
**Reactor 3**	351	299	299	290	288	285	284	285	280	275
**Reactor 4**	351	292	291	292	286	283	287	281	281	278
**Reactor 5**	351	300	284	283	285	287	275	287	272	267

Table S7. Record and process of the experimental data of COD in each of the five reactors during the ozonation process for inlet ozone concentrations of 25.8 mg L^{-1} in the five- reactor tandem ozone reaction devices.

Time (min)	0	15	30	45	60	90	120	150	180	210
**Reactor 1**	351	290	280	266	265	256	245	241	243	235
**Reactor 2**	351	289	276	270	250	251	246	245	240	236
**Reactor 3**	351	300	290	276	261	250	253	241	257	240
**Reactor 4**	351	296	291	276	262	253	250	246	250	236
**Reactor 5**	351	299	284	283	264	254	251	250	251	240

Table S8. Record and process of the experimental data of COD in each of the five reactors during the ozonation process for inlet ozone concentrations of 38.9 mg L^{-1} in the five- reactor tandem ozone reaction devices.

Time (min)	0	15	30	45	60	90	120	150	180	210
**Reactor 1**	351	280	251	247	233	220	196	200	196	201
**Reactor 2**	351	282	258	236	230	214	190	193	194	195
**Reactor 3**	351	299	253	241	238	221	206	201	203	197
**Reactor 4**	351	292	251	250	240	223	203	203	196	200
**Reactor 5**	351	290	254	246	236	230	202	203	198	200

Table S9. Record and process of the experimental data of COD in each of the five reactors during the ozonation process for inlet ozone concentrations of 59.9 mg L^{-1} in the five tandem ozone reactor.

Time (min)	0	15	30	45	60	90	120	150	180	210
**Reactor 1**	351	275	256	227	206	180	156	132	146	130
**Reactor 2**	351	270	251	220	200	174	153	132	133	130
**Reactor 3**	351	274	257	224	203	176	156	132	130	131
**Reactor 4**	351	274	255	224	205	174	153	140	141	130
**Reactor 5**	351	280	250	223	206	181	156	130	141	136

Table S10. Record and process of the experimental data of COD in each of the five reactors during the ozonation process for inlet ozone concentrations of 79.0 mg L^{-1} in the five tandem ozone reactor.

Time (min)	0	15	30	45	60	90	120	150	180	210
**Reactor 1**	351	268	245	210	181	156	127	120	113	117
**Reactor 2**	351	273	240	196	174	159	121	120	120	120
**Reactor 3**	351	272	250	206	180	155	128	123	112	112
**Reactor 4**	351	275	245	203	172	150	120	124	117	115
**Reactor 5**	351	269	250	213	170	160	121	126	115	113

(a) **(b)**

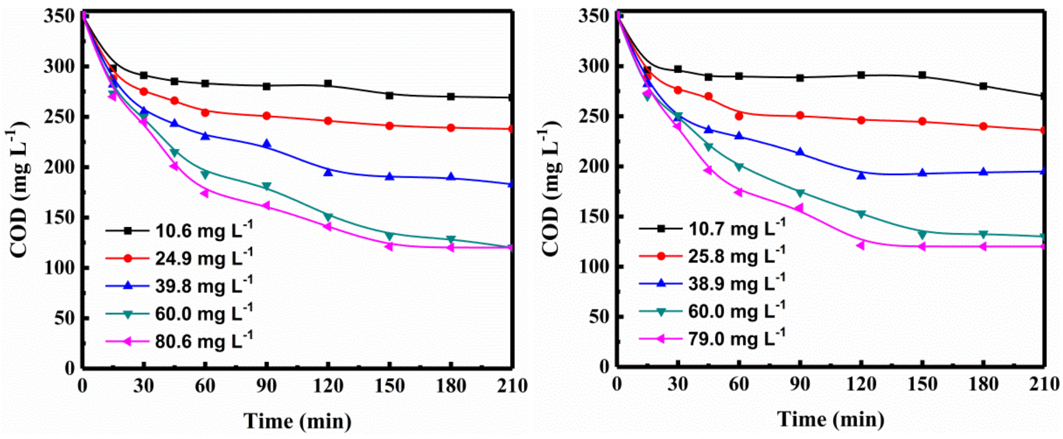

Figure S1. COD in the second stage of the (a) 3- and (b) 5-reactor devices at various ozone concentrations over 210 min.

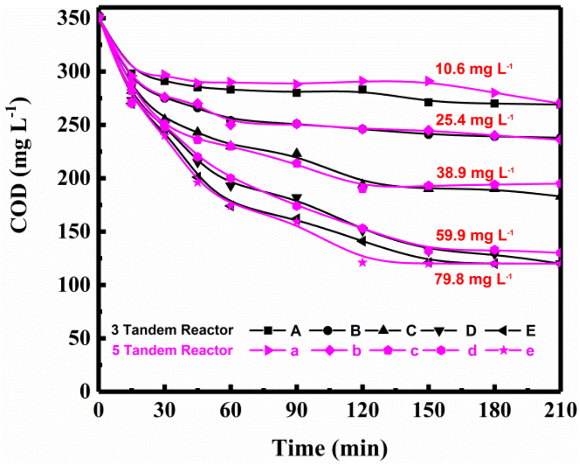

Figure S2. Comparison of COD in each of the second stages of the three- and five-tandem reactors at different levels of ozone concentration at 210 min.

Table S11. COD in the second stage of the 3- tandem reactor devices at various ozone concentrations over 210 min.

Time (min)	0	15	30	45	60	90	120	150	180	210
**10.6 mg L^{-1}**	351	298	291	285	283	280	283	271	270	269
**24.9 mg L^{-1}**	351	288	275	266	254	251	246	241	239	238
**39.8 mg L^{-1}**	351	282	255	243	230	223	194	190	190	183
**60.0 mg L^{-1}**	351	273	250	215	193	179	151	132	129	127
**80.6 mg L^{-1}**	351	270	245	201	174	159	141	121	120	120

Table S12. COD in the second stage of the 5- tandem reactor devices at various ozone concentrations over 210 min.

Time (min)	0	15	30	45	60	90	120	150	180	210
**10.7 mg L^{-1}**	351	296	297	289	290	288	291	291	280	270
**25.8 mg L^{-1}**	351	289	276	270	250	251	246	245	240	236
**38.9 mg L^{-1}**	351	282	258	236	230	214	190	193	194	195
**60.0 mg L^{-1}**	351	270	251	220	200	174	153	132	133	130
**79.0 mg L^{-1}**	351	273	240	196	174	159	121	120	120	120

Table S13. COD removal rate in the second stage of the three tandem reactors change with time at different ozone concentrations.

O_3 conc. (mg L^{-1})	COD removal %								
	15 min	30 min	45 min	60 min	90 min	120 min	150 min	180 min	210 min
**10.6**	14.6	17.5	19.1	19.9	20.9	21.1	23.5	23.8	24.2
**24.9**	16.2	19.7	23.2	25.5	27.2	30.0	31.2	31.7	32.3
**39.8**	18.8	26.4	31.1	33.7	37.4	43.5	45.0	45.4	46.1
**60.0**	21.1	28.1	37.7	45.1	49.1	56.9	62.3	63.3	63.9
**80.6**	22.5	30.2	40.4	49.6	53.7	59.2	65.0	65.7	66.4

Table S14. COD removal rate in the second stage of the five tandem reactors change with time at different ozone concentrations.

O_3 conc. (mg L^{-1})	COD removal %								
	15 min	30 min	45 min	60 min	90 min	120 min	150 min	180 min	210 min
**10.7**	14.9	16.0	17.7	18.6	19.4	19.3	18.9	21.4	22.3
**25.8**	16.0	19.0	21.9	25.8	28.0	29.1	30.3	29.3	32.4
**39.0**	17.8	26.6	30.5	32.9	36.9	43.2	43.0	43.8	43.4
**60.0**	21.8	28.4	35.7	44.9	48.4	57.1	62.4	62.1	62.9
**79.0**	22.7	29.9	41.4	50.3	54.7	59.9	65.6	65.8	65.7

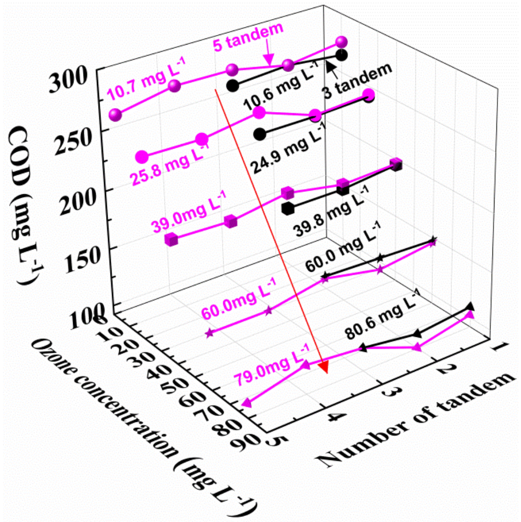

Figure S3. The COD at different ozone concentrations change with the 3- and 5-tandem reactors in 180 min.

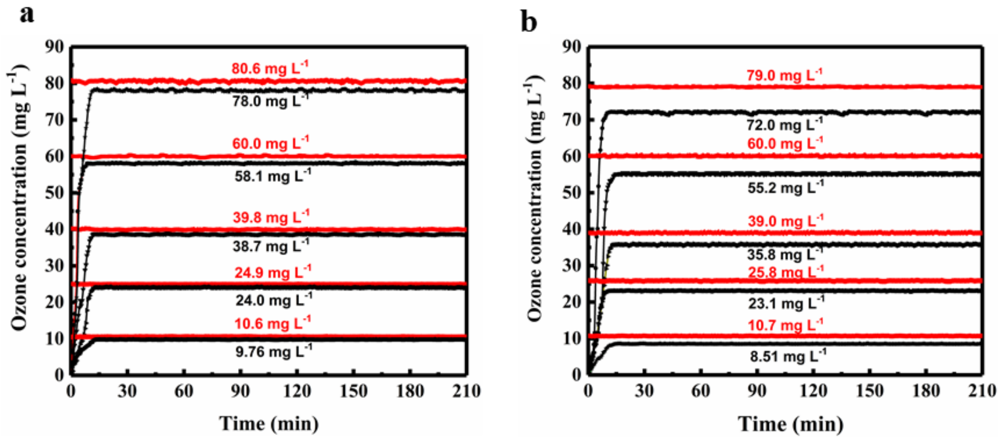

Figure S4. Ozone concentration at the inlet (red color line) and outlet (black color line) of the (a) 3-reactor and (b) 5-reactor tandem devices.

Table S15. Calculation of the consumption (%) and Ozone utilization efficiency (%) of the three-reactor tandem devices at 180 min.

Inlet O_3 conc. (mgL^{-1})	Outlet O_3 conc. (mg L^{-1})	Tot. O_3 consumption (mg)	ΔO_3 conc. (mg L^{-1})	O_3 consumption %	∑each tandem COD (mg L^{-1})	O_3 utilization efficiency %
79.0	72.2	136	6.83	8.65	1224	21.8
59.9	55.1	104	4.79	7.99	1016	11.7
38.9	35.7	67.2	3.24	8.32	762	11.4
25.8	23.0	44.0	2.82	10.9	514	10.0
10.7	8.51	17.3	2.19	20.5	190	9.01

Table S16. Calculation of the consumption rate (%) and Ozone utilization rate (%) of the five - reactor tandem devices at 180 min.

Inlet O_3 conc. (mg L^{-1})	Outlet O_3 conc. (mg L^{-1})	Tot.O_3 consumption (mg)	Δ O_3 conc. (mg L^{-1})	O_3 consumption %	∑each tandem COD (mg L^{-1})	O_3 utilization efficiency %
80.6	77.9	142.7	2.74	3.40	731	5.12
60.0	58.1	106.3	1.95	3.25	599	5.64
39.8	38.7	70.7	1.10	2.77	485	6.86
24.9	24.1	44.0	0.790	3.19	334	7.58
10.6	9.79	18.3	0.770	7.27	130	7.10
